# A non-invasive biomechanical model of mild TBI in larval zebrafish

**DOI:** 10.1371/journal.pone.0268901

**Published:** 2022-05-27

**Authors:** Carolina Beppi, Marco Penner, Dominik Straumann, Stefan Yu Bögli

**Affiliations:** 1 Neuroscience Center Zurich, University of Zurich and ETH Zurich, Zurich, Switzerland; 2 Department of Neurology, University Hospital Zurich and University of Zurich, Zurich, Switzerland; 3 Clinical Neuroscience Center, University Hospital Zurich and University of Zurich, Zurich, Switzerland; 4 Swiss Concussion Center, Schulthess Clinic, Zurich, Switzerland; University of Florida, UNITED STATES

## Abstract

A mild traumatic brain injury is a neurological dysfunction caused by biomechanical forces transmitted to the brain in physical impacts. The current understanding of the neuropathological cascade resulting in the manifested clinical signs and symptoms is limited due to the absence of sensitive brain imaging methods. Zebrafish are established models for the reproduction and study of neurobiological pathologies. However, all available models mostly recreate moderate-to-severe focal injuries in adult zebrafish. The present work has induced a mild brain trauma in larval zebrafish through a non-invasive biomechanical approach. A custom-made apparatus with a commercially available motor was employed to expose larvae to rapidly decelerating linear movements. The neurophysiological changes following concussion were assessed through behavioural quantifications of startle reflex locomotor distance and habituation metrics. Here we show that the injury was followed, within five minutes, by a transient anxiety state and CNS dysfunction manifested by increased startle responsivity with impaired startle habituation, putatively mirroring the human clinical sign of hypersensitivity to noise. Within a day after the injury, chronic effects arose, as evidenced by an overall reduced responsivity to sensory stimulation (lower amplitude and distance travelled along successive stimuli), reflecting the human post-concussive symptomatology. This study represents a step forward towards the establishment of a parsimonious (simple, less ethically concerning, yet sensitive) animal model of mild TBI. Our behavioural findings mimic aspects of acute and chronic effects of human concussion, which warrant further study at molecular, cellular and circuit levels. While our model opens wide avenues for studying the underlying cellular and molecular pathomechanisms, it also enables high-throughput testing of therapeutic interventions to accelerate post-concussive recovery.

## Introduction

### Mild brain trauma–definition and characteristics

A concussion, or mild traumatic brain injury (TBI), is a transient neurological dysfunction caused by biomechanical forces transmitted to the brain with a direct blow of the head, or indirectly, due to inertial loading from impulsive motions of the body [1,2]. According to the U.S. Centers for Disease Control and Prevention, concussion is the most recurring form of TBI (75%) and constitutes a major health concern worldwide, with an estimated incidence of 1-6/1000 affected annually [3–6]. Concussion results in a variety of clinical signs and symptoms, including headache, dizziness, impaired concentration, sleep disorders, blurred vision, hypersensitivity to light or noise, imbalance, confusion, incoordination and nausea [7,8]. In some cases, concussion leads to transient loss of consciousness [9]. Current theories suggest that, upon a head impact, the brain undergoes a metabolic shock characterized by the perturbation of ionic influx across neural cell membranes and decreased energy disposal for restoring the ionic disruption [10]. The metabolic function usually restores within approximately 7 days [11,12], along with the resolution of the acute clinical signs and symptoms [13]. Recently, also blood-brain barrier disruption has been demonstrated in mild TBI [14]. However, repeatedly concussed individuals [15], young adults and children [16–18] may incur longer lasting consequences [9].

The post-concussive clinical profile is highly heterogeneous depending on the severity [19] and the biomechanics [9,20] of the impact. The forces acting on the brain as a result of an impact have a linear acceleration (LA) and an angular acceleration (AA) component [21–23]. Their relative magnitude is defined by the distance of the impact’s force vector from alignment with the head’s longitudinal axis, with the LA/AA fraction being larger the closer the alignment [24]. Tissue injuries can result from both LA and AA forces [25], while structural and functional disruptions at the intracellular level are due to LA forces [26,27]. As most impacts occur off alignment of forces and head longitudinal axis, they involve a mixture of AA and LA forces [28].

In contrast to more severe forms of TBI, concussion does not lead to focal injuries (e.g., bruising or bleeding) visible on standard brain-imaging methods, such as computed tomography, positron emission tomography, and MRI [29,30]. The absence of sufficiently sensitive imaging methods and the significant heterogeneity of cases has challenged a complete understanding of the neuropathological biochemical events following concussion in humans [29].

### The potential of animal model organisms

Animal models constitute a useful tool to reproduce and study in a controlled and detailed mode neuropathological processes underlying different neurological diseases. Zebrafish have become an established animal model due to several advantages, including the limited size and numerous offspring, the easy breeding and fast development [31,32]. Zebrafish are particularly suitable for neurobiological studies as, like other non-mammalians, they have a structurally simpler neural circuitry compared to humans, yet preserving several functional features at the behavioural, cellular and molecular levels [33–39]. Robust microscopic imaging, microsurgical and optogenetic approaches can be implemented combined with genetic manipulations or/and pharmacological interventions to study the relationship between genes, brain structure/function and behaviour [40–45].

Currently available brain lesion models in zebrafish include stab lesion assays [46,47], weight-drop models (e.g., [48]), open head models [49], mechanical or neurotoxic cerebral injuries via high-intensity focused ultrasound [50], microinjection of immunogenic particles [51] or excitotoxic quinolinic acid [52]. These models represent valuable experimental simulations of moderate to severe forms of TBI. They are, however, invasive, only applicable with adult zebrafish and cannot translate to mild TBI forms, namely those not resulting in focal injuries. Larval zebrafish models would be especially valuable due to several advantages. In particular, the simple cerebral structure and the optical translucency in larval stages [31,43,44] allow detailed and non-invasive microscopic screenings of the pathological CNS changes over time, at the cellular and metabolic levels.

### Aims and hypotheses

This study aimed at recreating concussion in larval zebrafish through a non-invasive biomechanical approach to study the CNS changes caused by linear deceleration forces. To such purposes, a custom-made apparatus with a commercially available linear motor was used to expose larval zebrafish contained in a liquid-filled capsule to a rapid earth-vertical linear movement with a first slow acceleration followed by an abrupt final deceleration (peak: 385 m/sec^2^). The behavioural changes following concussion were assessed through behavioural quantifications of the acoustic/vibratory startle reflex habituation (SRH), based on the analytical approach of Beppi et al. [53,54]. Impaired SRH modulation characterise several neurological and psychiatric conditions in both zebrafish and humans, including TBI [55], schizophrenia [56–58], autism [58] and post-traumatic stress [59,60]. As such, the SRH is considered an important index of neurophysiological health for clinical and translational investigation. Healthy larval zebrafish were randomly assigned to two groups (control or concussion) and tested at baseline to obtain a normative behavioural profile. After the concussion group underwent the impact, both the groups were retested at five post-injury (P-I) times to assess eventual between-groups differences across time. We predicted that the groups would be behaviourally indistinguishable at baseline, but that their SRH performance would instead differ significantly at different P-I times.

## Materials and methods

### Ethical approval

All experiments were performed in accordance with the animal welfare guidelines of the Federal Veterinary Office of Switzerland (ZH190/2020, 32971). The committee reviewed and approved the eventual mortality of tested animals in this study.

### Fish maintenance, mating and egg production

Wild-type adult WIK *danio rerio* zebrafish lines were fed and maintained in line with standard protocols. Their embryos were incubated and raised under a 14-10-hours light-dark cycle in 28°C in 1.7L transparent breeding tanks. At 3–4 days past fertilization, they were transferred to 35x12 mm cell culture plates with E3 medium (solution in mM: 5 NaCl, 0.17 KCl, 0.33 CaCl_2_, and 0.33 MgSO_4_; Sigma-Aldrich Corp., St. Louis, MO, USA) and maintained there until the start of the experiment the following day.

### Experimental apparatus

**Fig 1:**
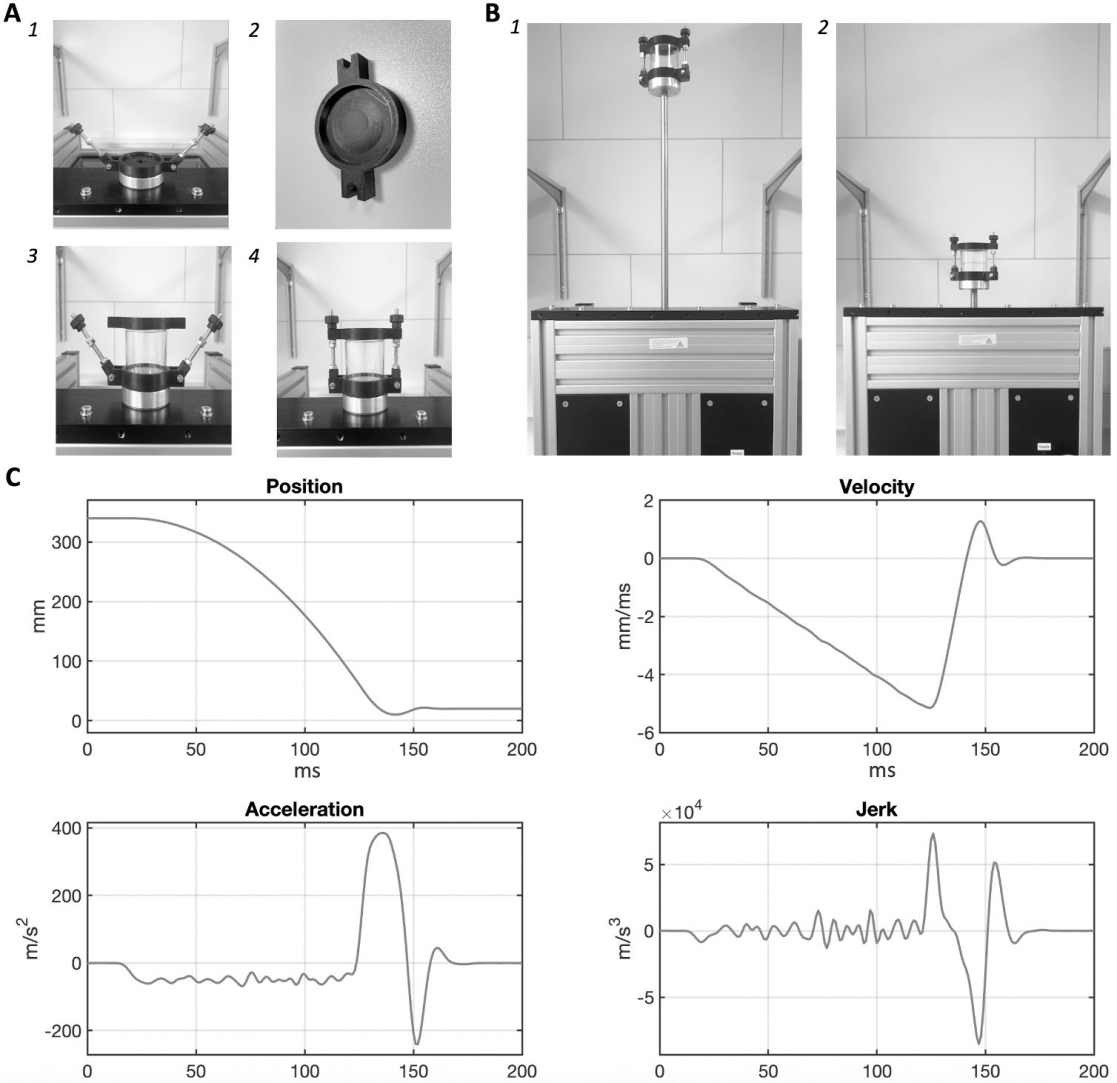
Concussion procedure. (A) Illustration of the mounting of the cylindrical capsule (containing fish in water) on the holder, following the numerical order of the panels. The capsule’s black lid was provided, on its side exposed to water, of a convex rounded protuberance of smaller diameter than the cylindrical capsule (2). This shape allowed any air contained in the capsule to be expelled by pressure when the lid was placed on top of the capsule (containing fish in water). The lid-covered capsule was placed on the holder, which had a cylindrical cavity (size-matching the capsule’s diameter) that prevented the capsule from moving side-wise (A1-A3). The screws on the sides were then used to securely lock the capsule (3,4). (B) Illustration of the movement performed by the motor: the capsule is slowly brought up to the start position (B1) and rapidly slides down to the end position (B2) with the parameters described in the following panels. (C) Features of the linear movement plotted as a function of time (ms): position, velocity, acceleration and jerk.

The concussion apparatus consisted of a motor device (model S01-72/500, LinMot, Spreitenbach, Switzerland) with a static part (stator, model: PS01-23x160H-HP-R) that was mounted and fixed on an impact-absorbing stone table. The moving component (slider, model: PL01-12x480/440-HP, diameter: 12mm; length: 480mm) could slide in upwards/downwards direction passing through a constitutive fissure within the stator. A small cylindrical holder with a central circular cavity was customised in 3D-printing and fixed on the tip of the slider to accommodate and hold the capsule (containing the fish) during the movement executions. The entire setup is depicted in Fig 1A and 1B. The motor device was paired and controlled by an inbuilt software (LinMot-Talk 6.9), which fed the motor with an input expressed in change in position (mm) over time (sampling rate: 0.001). The Servo Drive we used was a C1250-LU-XC-OS-000. The input was created using an inbuilt software library (Create Curve, Limited Jerk), which designed the motion curve based on the desired limit parameters: start point (+/-mm) and end point (+/-mm) relative to the home position, maximal velocity (m/s), maximal acceleration (m/s^2^), maximal deceleration (m/s^2^) and maximal jerk (m/s^3^). It was thereby obtained a motion curve with the following features: duration (~121 ms), length (320 mm), peak velocity (~5.1 m/s^2^), peak (initial) acceleration (~61 m/s^2^), peak deceleration (~385 m/s^2^), illustrated in Fig 1C. Several pilot-runs with small samples were run–before this study–to ensure that the motion curve would not cause the death of the fish within 48h in at least 90% cases. The software was provided with a built-in digital oscilloscope that could obtain a readout of a motion curve being performed, stored as csv-file. The oscilloscope was used to ensure the reliability of the apparatus in repeated executions of the same motion curve. The triggering input commands in the software (home & start) were connected, for convenience, to manual actuators: a red button (home) and a green button (start) on the protective chamber.

### Experimental procedure

On the testing day, zebrafish larvae of 92–96 hours past fertilization (N = 48) were divided and randomly assigned to either the concussion (N = 24) or the control (N = 24) condition:

1. The concussion group was transferred into a transparent polystyrene cylindrical capsule (48x52 mm, 60 ml), filled with E3 medium and air-free. The capsule was fixed on top of the slider, and the motor device was activated. Upon manual press, the motor would perform the stroke (see Fig 1A and 1B). After the concussion, the fish were inspected individually. The criteria for inclusion in the study were: (1) absence of physical damage (e.g., bending); (2) the ability to move freely without impediment. All fish passing this visual inspection would be subsequently transferred into a 24-well plate and assessed for their 5 minutes P-I test. Any fish failing the inclusion criteria would be withdrawn from the study and checked for whether they reached the humane endpoints specified in the following section.
2. The control group was transferred to another transparent polystyrene cylindrical capsule filled with E3 medium (identical to the concussion group; shown in Fig 1A3-4) for the whole duration of the mechanical injury of the concussion group. This ensured that the two groups would be exposed to the same handling, the same environmental conditions, for the same amount of time, minimising potentially confounding effects unrelated to the experimental manipulation, such as increased anxiety and arousal states related to a changed environmental setting (see Yokogawa et al. [61] and Bai et al. [62]). After concussion of the other group, the control fish were transferred into a 24-well plate–identical to that of the concussed group–and contemporaneously assessed for their 5 minutes P-I test.

The two groups (concussed; healthy control) would be assessed in parallel again 30 minutes, 1 hour, 5 hours, 24 hours after injury. Before each test, the fish would be again inspected to check for adherence to the same inclusion criteria, or for whether any fish instead reached the humane endpoints (specified in the following section). In between each test repetition, the fish (both groups) were kept in incubation in their respective 24-wells plates, under the same conditions, until the final 24 hours P-I test on the following day.

This procedure was performed three times on different days (sessions), at the same time of the day. On each session, 24 fish were concussed and tested in parallel with 24 control fish. The results of the three sessions were added together, reaching a *N* size of 72 fish (24 fish x 3 sessions) in each group (concussed; healthy control).

### Humane endpoints

All the fish failing the inclusion criteria for the study would be immediately assessed for whether they approached humane endpoints. The score is marked on the position on the plate where the animal is held. Swimming behaviour is assessed within two minutes. During early larval stages, zebrafish are not particularly motile unless there is a direct incentive (e.g., flight response to danger or startling stimuli). This is largely due the unnecessity of food, which is in fact provided by the remaining yolk until the end of day 7. As such, tapping the dish is a simple and easy way to test activity. The score is given as binary outcome: 0 (if no movement occurs, even after stimulation using tapping) or 1 (if movement(s) occur spontaneously or after dish tapping). All animals not reaching a score of at least 1 within two minutes would be euthanised.

### Behavioural paradigm

The experimental paradigm consisted of a series of 20 consecutive identical side-wise vibratory pulses (300-Hz, 126-dB) with instantaneous onset/offset (step pattern). Each stimulus lasted 500-ms and was interleaved by inter-stimulus intervals (ISI) of 500-ms (total duration = 31 sec). The paradigm was adapted from a recent work by Beppi et al. [53].

### Behavioural tracking and quantitative modelling

The locomotor activity of the fish upon vibratory stimulation was tracked through the Viewpoint Zebrabox (ViewPoint Life Sciences, Lyon, France) behavioural recording system and software. The speaker, designed and produced in 3D printing by ViewPoint, was positioned at a 11 cm distance from the centre of the well and used, together with a commercial amplifier (CS-PA 1 MK, Dynavox), to produce side-wise vibratory stimuli of the specified power (dB) and frequency (Hz).

Startle responses were quantified as total distance travelled (mm) occurring within the duration of each single stimulus (500-ms). Eventual movements performed during the ISIs were not quantified. The locomotor distance travelled at each stimulus was computed for each fish and the group’s TDT_20_ –computed by averaging the TDT_20_ from each individual fish–was statistically tested for eventual differences between the experimental conditions across 6 test times: pre-injury (baseline), 5 minutes, 40 minutes, 70 minutes, 5 hours and 24 hours P-I. The earliest three test times were set to track eventual changes in behaviour in the acute phase: immediately after injury (5-min), 30 min P-I–namely the time by which eventual loss of consciousness typically recovers in humans [63]–and 1 h P-I. The last two test-times were set to track eventual long-term effects: an early chronic phase (5 h P-I) and a late chronic phase (24 h), namely the time concussed individuals normally require to resolve their acute signs/symptoms [64].

Only fish that survived without displaying physical/motor injury and performed all 6 tests were included in the analyses. Data bootstrapping procedure (N = 500, in-built MATLAB function: bootstrp) was implemented on the habituation curve (distance travelled over stimuli) of each individual fish (of both groups) to obtain a population-level distribution of behaviour. A first-order exponential (in-built MATLAB function: lsqcurvefit) was fitted into the bootstrapped data (N = 500)–using the SRH model proposed by Beppi et al. [53,54]–obtaining three descriptive measures of startle habituation from each bootstrap. The *amplitude* is defined as the ‘vigorousness’ of startle reflex, in terms of distance travelled in reaction to the first stimulation. The *offset* is the steady-state response level (as distance travelled per stimulus) obtained in the long-term. In other words, the baseline responsivity of a fish after it has habituated to a repeated stimulus. The *decay constant* is the number of stimuli required for the amplitude to fall to 1/e (~ 36.8%) of its initial value.

### Definition of mild TBI in larval zebrafish

Some important factors that distinguish mild TBI from moderate-to-severe injuries are: (1) the usually negative CT/MR imaging, which in moderate/severe is instead diagnostic; (2) the absence of focal neurological signs, which in moderate/severe are frequently present; (3) CGS, where eventual loss of consciousness is none or transient, whereas in moderate/severe it is frequently present [63]. The movement kinematics of deceleration in our experiments were hence determined to induce a mild TBI based on the following aspects: (1) the presence of [transient] post-concussive neurological deficits in the absence of a physical/motor disability, (2) the recovery of acute neurological symptoms within 70-min P-I.

### Statistical analyses

Statistical analyses were conducted using MATLAB R2021a (The MathWorks Inc., Natick, Massachusetts, USA) and SPSS Statistics version 27.0 (IBM Corp., Armonk, New York, USA). A 2x6 mixed-design ANOVA was then performed to assess potential differences in mean TDT_20_ between the groups (control fish versus concussed) and across different test times. The dependent variable consisted of the mean TDT_20_, as continuous measure. The first independent variable consisted of the two independent groups (concussion or controls), while the repeated-measures IV was time, with 6 levels: pre-injury (baseline) and the 5 P-I test-times (5 minutes, 40 minutes, 70 minutes, 5 hours and 24 hours P-I).

## Results

### First inspection after concussion

A water-filled capsule containing freely swimming zebrafish larvae was linearly accelerated and decelerated with a peak deceleration of 385-m/s2, i.e., 39.3g (Fig 1). After such one-time concussion, all exposed larvae survived and passed the visual inspection, showing no motor impediments resulting from a physical damage.

### Startle response habituation before and after concussion

**Fig 2:**
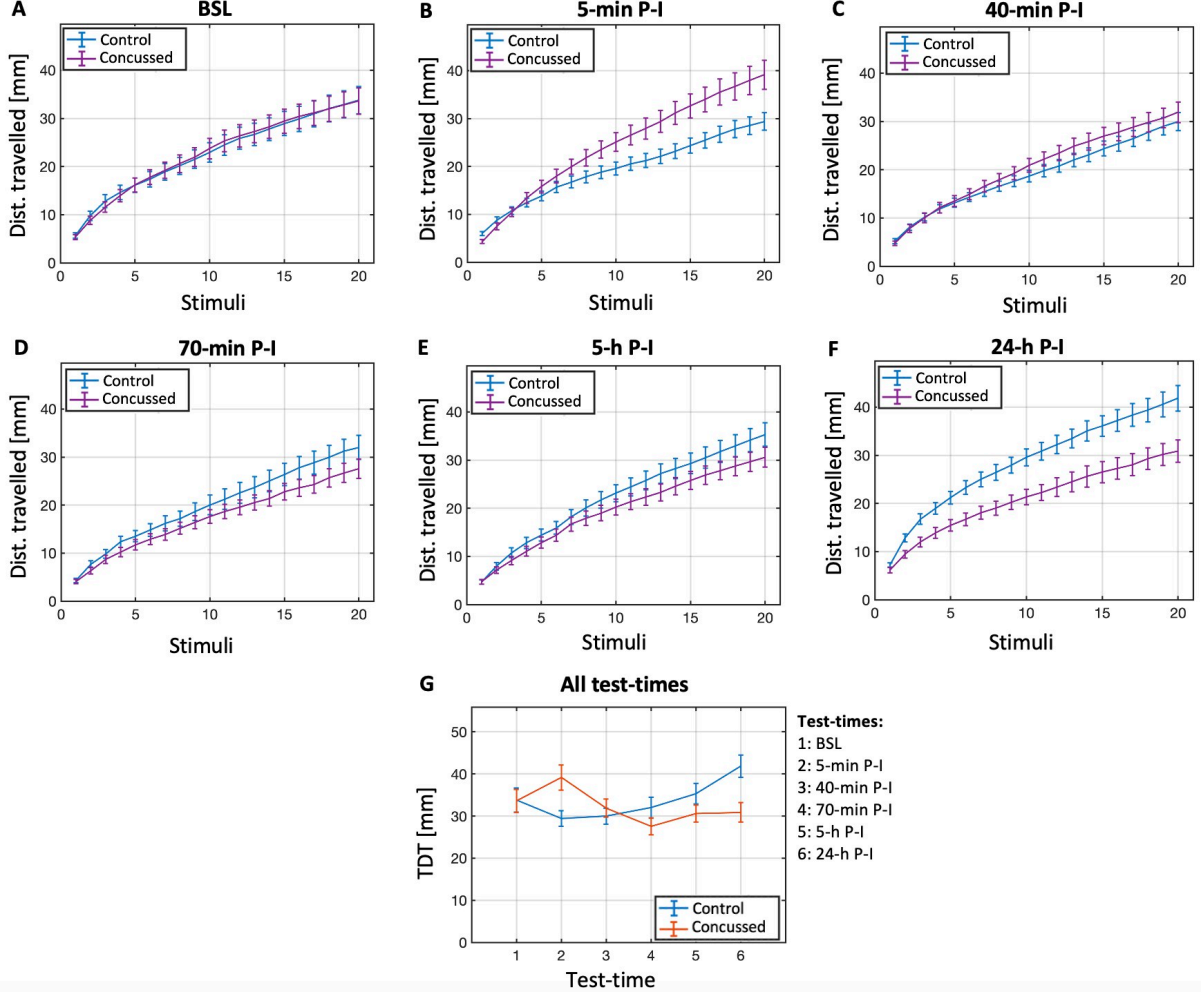
Startle responses over test-times. Mean distance travelled (+/-1 SE) at each stimulus, for both groups (N = 72, each), at each time point (A-F). Total distance travelled after 20 vibratory stimuli (M +/-1 SE) for both groups (N = 72, each) at each time point (G). A 2x6 mixed ANOVA (N_control_ = 72, N_concussed_ = 72) was run. No significant main effect of group (F(1,142) = 0.499, p>0.05) on the mean TDT_20_ was found, with controls (M = 33.724, SE = 1.446) and concussed (M = 32.28, SE = 1.446) performing similarly overall. A significant main effect of time (F(4.365,619.792) = 2.559, p = 0.033) was observed, although without significant differences at the pairwise level. The effect of time was different for the control and concussed group (G), as confirmed by a significant interaction between group and time (F(4.365, 619.792) = 5.644, p<0.001). The interaction effect was then broken down through two one-way repeated-measures ANOVAs, which analysed the effect of time (six levels) for the control and concussed groups separately. For the control group (N = 72) there was a significant effect of time (F(4.137,293.712) = 4.825,p<0.001). Pairwise comparisons further indicated that the mean TDT_20_ was significantly higher at time 6 (24-h P-I, M = 41.885, SE = 2.655) than at time 2 (5-min P-I, M = 29.406, SE = 1.859) with p<0.001, at time 3 (40-min P-I, M = 29.975, SE = 1.898) with p = 0.004. A significant effect of time (F(4.069,288.933) = 3.403, p = 0.009) was found also for the concussed group, whose mean TDT_20_ was significantly lower at time 4 (70-min P-I, M = 27.547, SE = 2.018) compared to time 2 (5-min P-I, M = 39.142, SE = 3.027) with p < .001. No additional statistical differences at pairwise levels were observed.

We tested SRH before and after concussion. At different time points after the concussion, trajectories of the cumulative distance travelled over 20 vibratory stimuli showed distinct differences of the concussed larvae (N = 72) compared to the non-concussed (control, N = 72) larvae (Fig 2): 5 minutes after concussion, the total distance travelled after 20 stimulations (TDT_20_) was significantly larger in the concussed group (Fig 2B and 2G test-time 2), while one day later the TDT_20_ it was significantly smaller (Fig 2F and 2G test-time 6). Note that the TDT_20_ of the control group increased significantly over 24 hours. Statistical numbers are provided in the Fig 2 legend.

### Exponential fitting of cumulative distances travelled

**Fig 3:**
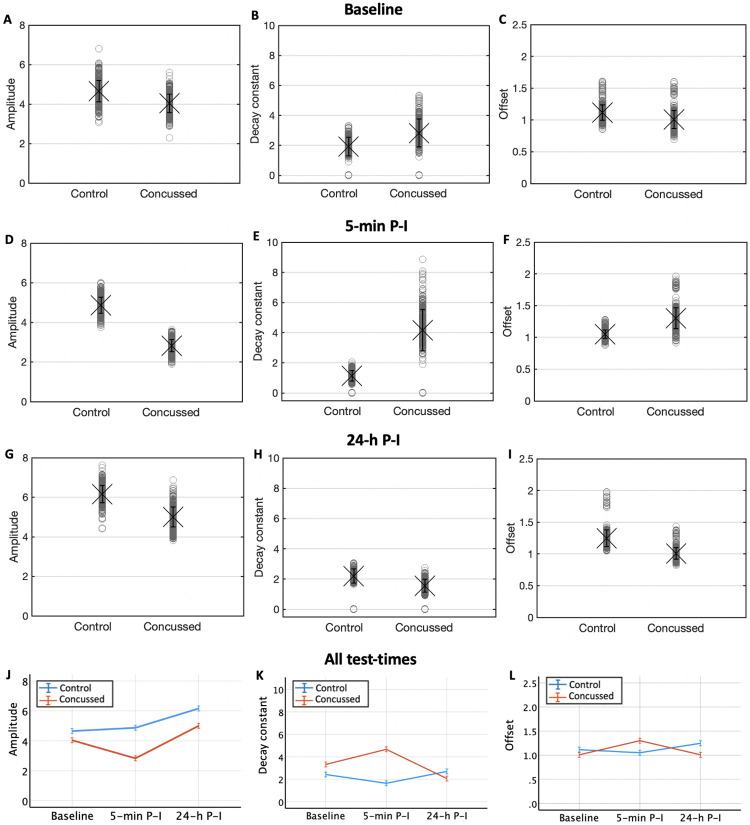
Population-level habituation descriptives. Descriptive statistics for the mean (+/- 1 SE) amplitude (A, D, G), decay constant (B, E, H) and offset (C, F, I) of 500 bootstraps, for both groups (control and concussed), at baseline, 5 minutes and 24 hours P-I. Change (mean +/- 2 SE) in amplitude (J), decay constant (K) and offset (L) over the same 3 test-times.

By fitting the habituation curves (distances travelled over subsequent 20 stimulations) of 500 bootstraps to a single exponential, we explored which parameter of SRH (amplitude, decay constant, offset) were responsible for the differences between concussed and non-concussed larvae (Fig 3). Five minutes after the injury, the increased TDT_20_ of concussed larvae was largely due to an increased decay constant (Fig 3E and 3K), despite decreased amplitude (Fig 3D and 3J). One day (24-h) after concussion, the decay constant had normalized (Fig 3H and 3K) although the amplitude (Fig 3G and 3J) and offset (Fig 3I and 3L) were smaller compared to that of the control group, thus concussed larvae were generally less reactive upon individual stimulations.

## Discussion

Concussion is a neurological dysfunction of major concern worldwide. The heterogeneity of impact profiles, clinical signs and recovery times still challenge a univocal definition and diagnosis of concussion. Furthermore, a full understanding of the underlying pathological bio-chemical events is still elusive due to the absence of sufficiently sensitive imaging methods. Animal models offer the possibility of experimentally recreating specific brain pathologies to study the neural underlyings in a controlled manner. Zebrafish are particularly suited for neurobiological studies due to the functional similarities with the mammalian brain, as well as the simple breeding and maintenance of large stocks. However, currently available zebrafish models are invasive, only applicable with adult zebrafish and fail to translate to mild TBI forms that do not involve a focal structural injury.

This study aimed at establishing a non-invasive model of concussion in larval zebrafish via biomechanical transmission. Larval zebrafish were enclosed in an air-free capsule filled with E3 medium and exposed to a rapid decelerating earth-vertical movement using a custom-made apparatus with a commercially available linear motor. Behavioural changes after concussion were assessed through quantifications of SRH, based on the methodology of Beppi et al. [53,54]. The fish were tested at baseline, and again at five different P-I times against a control group, to evaluate the behavioural consequences of concussion over time. It was hypothesised that the groups’ behaviour would not differ significantly at baseline and that they would instead display significantly different SRH performance at the different P-I times.

The concussed and control groups behaved similarly at baseline, displaying an almost identical total distance travelled after 20 stimulations (TDT_20_), suggesting that no intrinsic behavioural differences between groups were present before concussion. The control group showed a TDT_20_ increase of over the course of 24 hours, reflecting the naturally marked developmental growth of larval zebrafish in their first few days of life. In contrast, the concussed group showed a pattern of TDT_20_ reduction over time, evidencing the existence of a chronic neurological dysfunction, in otherwise healthy fish. Strikingly, at 5 minutes P-I, the concussed group displayed a little-to-null habituation, which–despite the decreased amplitude of the exponential fit–resulted in an increased TDT_20_.

The proposed SRH model–described by Beppi et al. [53,54]–relies on the observation that the consecutive decrease of locomotion distance with each stimulus can best be quantified by a single decay constant. The smaller the decay constant, the more effective the habituation; the larger the decay constant, the weaker the habituation. An infinitely large decay constant indicates the absence of habituation. The decay constant is independent of the amplitude of the initial startle response (amplitude—offset) or the steady-state startle response after the effect of habituation (offset). For instance, a strongly decreased amplitude combined with a slightly increased offset can still go together with an enlarged decay constant, i.e. with impaired habituation (such is the case of the concussed group, 5-min P-I). Similarly, a high overall response to all 20 stimuli (TDT_20_), does not necessarily reflect weak habituation, but can be the result of a strong steady-state response (offset) as well.

The increased TDT_20_ along with lack of habituation (incl. low amplitude, high offset) might be symptom of a transient neurological deficit induced by the mechanical injury and of anxiety (high arousal) related to exposure to environmental stressors [61,62]. Interestingly, the behavioural observations mirror the common acute post-concussive symptom of hypersensitivity [7]. It seems however unlikely that only psychogenic factors are in play, in that–as we will discuss later–the fish showed chronic signs of deficit (decreased TDT_20_) still after 24-h–a time by which anxiety and arousal levels restored to baseline. This suggests the occurrence of a CNS/neurological dysfunction beyond the effect of induced anxiety and arousal states.

A possible way for future experiments to monitor the effect of the anxiety factor would be to assess serotonergic (dorsal raphe—optic tectum) function/activity following concussion using calcium imaging. Eventual behavioural differences between groups could then be adjusted for by any differences in serotonergic activity (see Yokogawa et al. [61] and Bai et al. [62] for signatures and measures of anxiety). This would ensure a more direct relation of (eventual) behavioural differences to the mere mechanical exposure.

Acute P-I signs largely recovered in the successive 40 minutes, as evidenced by TDT_20_ of the concussed group returning close to control levels. In line with this, no differences in amplitude, decay constant or offset were found at the population level. At 70 minutes P-I, signs of chronic effects started to be noticeable, with the concussed fish showing a slightly lower amplitude and mean distance travelled across the stimuli compared to controls. These behavioural patterns still held until 5 hours P-I. However, no differences in amplitude, decay constant or offset were (yet) visible at the population level.

One day after injury, the concussed fish, compared to controls, displayed a markedly lower amplitude and mean distance travelled across the stimuli, mirroring the post-concussive syndrome often affecting a significant group of concussed individuals [65]. Indeed, despite concussion is traditionally described as a temporary and reversible brain dysfunction, it is frequently associated with long-lasting post-concussive symptoms and functional disturbances [66]. Despite the overall reduced responsivity to sensory stimuli at 24 hours P-I, the habituation seemed to be preserved. This could suggest that states of anxiety and high arousal might indeed have been responsible for the lack of habituation within the 5-min P-I [61,62]. On the other hand, the recovery might be a result of the extraordinary CNS plasticity of which larval zebrafish, and non-mammalians in general, are capable [33–35,67]. “Plastic” neural re-organisations may have restored brain functioning despite cellular (e.g., metabolic) or molecular deficiencies. Indeed, humans with persistent post-concussive syndrome (PCS) show abnormal brain activation patterns [68,69], while those who recover from the PCS display frontal hyperactivations during executive function tasks [66,70]. This suggests that mechanisms of functional plasticity might compensate for long-term post-concussive brain dysfunctions, restoring their cognitive abilities. Connectivity analyses would be extremely valuable to test this hypothesis. Intracellular regeneration is another recovery process that might have contributed, and cannot be excluded. It would hence be important, as a next step, to consider assessing several metabolic processes, including mitochondrial transport [71] and mitochondrial responses during cell stress conditions [72], as well as the action of macrophages [73].

While the behavioural changes/deficits observed in larval zebrafish are promising, further experimentation would be necessary to understand the underlying neurobiological alterations, as well as the structural changes that may mediate recovery. At the RNA level, one might consider studying the expression of genes that drive the injury response at cellular and molecular levels. Immunostainings could instead be performed to track cell death and eventual neural regeneration. Importantly, as intracellular regeneration cannot be excluded, western blotting with appropriate markers (e.g., Caspase 3) might also be considered.

## Conclusions

Our biomechanical model applying short-term linear forces on zebrafish larvae enclosed in water-filled capsules–despite limitations which we will discuss in the next sections–represents a step forward towards the establishment of a parsimonious behavioural model of mild TBI (i.e., simple/basic, with low ethic burden, yet sensitive to the experimental manipulation). The mechanical injury in fact induces different physiological-behavioural responses, which warrant further investigation at molecular and cellular levels (through analyses of gene expression, immunostainings and western blots) to elucidate the structural and functional changes that underly the observed behavioural effects, and the functional recovery response.

Our results indicate that the decay constant of startle habituation in larval zebrafish can represent a sensitive index of acute CNS dysfunction following concussion, while deficits of motor reactivity upon sensory stimulation, in the absence of abnormal habituation, may be indicative of long-term/chronic effects mirroring human post-concussive syndrome.

We consider some methodological limitations in this study and discuss aspects that can be considered for further investigation. First, we acknowledge that the biomechanical transmission of forces was not selectively affecting the head/brain, but the entire body. It follows that central and peripheral elements of the motor system might have been also affected, producing the observed behavioural deficits. Several reasons make this possibility seem unlikely. Since the brain is less protected at larval stages (no skull, little fluid around the brain, it is arguably the most vulnerable organ. This hypothesis is supported by no evidence of motor alterations or/and physical damage found upon close inspection of the fish after concussion. Additionally, the existence of a physical/motor injury would not allow the acute effects to resolve within 5 hours, as the tissue regeneration times in larval zebrafish are longer [74]. Therefore, we conclude that the observed deficits are of central nature.

Furthermore, as the dynamics of force transmission by impacts in liquids (e.g., cells) are different from those in the atmosphere, the validity and translational value of the model might be questioned. However, it could be considered that zebrafish larvae enclosed in water capsules might represent valid proxies of neural populations within the brain. Their behavioural alterations might provide relevant insights onto the cellular and molecular neural dysfunctions. While this biomechanical model opens avenues for studying the neurobiological pathomechanisms that follow a concussion in larval zebrafish, it also enables the assessment of the effect of therapeutic interventions apt to facilitate post-concussive recovery.

Future investigations could design multiple movement profiles to comparatively assess the effects of stronger and milder linearly accelerating/decelerating impacts, to ultimately build a ‘tolerance curve’. This would be descriptive of the critical ‘cumulative stress’ (in terms of liner mechanical forces) the brain can be exposed to, before incurring an irreversible, chronic or/and neurodegenerative damage. Importantly, having multiple treatment groups exposed to different curves of movements would be a straightforward way to control (balance out) the effect of induced anxiety across groups. In this context, it is known that repeated concussions in sports have been shown to result in more severe consequences [75–77]. Thus, one might consider exploring the effect of repeated sub-concussive impacts in zebrafish, like in rats (e.g., [78,79]), re-executing the same linear movement at different time intervals from the first. The results of such experiment would be valuable to define the critical time window of high vulnerability during which the brain would register cumulative damage.

Ultimately, one would comparably assess the effect of linearly accelerating impacts and of movements involving increasingly angular acceleration forces [80], which have been associated to more distributed brain damage [24,25] and more severe diagnoses [77]. It would be useful to assess the state of other sensory-processing systems after concussion.

Importantly, behavioural screenings in additional sensory modalities, such as the visually induced SRH [81,82] the optokinetic reflex (e.g., [83,84]) and the vestibulo-ocular reflex (e.g., [85,86]) would be required for a more comprehensive understanding of the dysfunctions following our induced mTBI.

We consider our model as being a promising tool that requires further molecular and cellular understanding as to the injury and recovery responses that underlie altered behaviour. More investigation is required also to assess whether the model can be employed to assess the effect of therapeutic molecules on post-TBI recovery. The model can represent a step forward towards the reduction of the ethical burden of animal models. Arguably, exposure to non-lethal decelerations of zebrafish larvae has a marginal impact compared to other more invasive animal models of concussion (e.g., pharmacological, genetic manipulations, weight-drop apparatus, stab lesions, head-piercing). We encourage future animal work for translational purposes to maintain the ethical burden as low as possible.

## Abbreviations

AA: angular acceleration
BSL: baseline
LA: linear acceleration
ISI: inter-stimulus interval
TBI: traumatic brain injury
PCS: post-concussive syndrome
P-I: post-injury
SRH: startle reflex habituation
TDT: total distance travelled
